# Challenges Associated with the Implementation of an Exercise and Sport Intervention Program in a Greek Refugee Camp: A Report of Professional Practice

**DOI:** 10.3390/ijerph16244926

**Published:** 2019-12-05

**Authors:** Florian Knappe, Flora Colledge, Markus Gerber

**Affiliations:** Department of Sport, Exercise and Health, University of Basel, 4052 Basel, Switzerland; florian.knappe@stud.unibas.ch (F.K.); flora.colledge@unibas.ch (F.C.)

**Keywords:** acceptability, European migration crisis, Greece, refugee camps, sport and exercise interventions, sustainability

## Abstract

*Objective* Refugees have a particularly high prevalence of psychopathological disorders. Despite this, little attention has been paid to the treatment of traumatized refugees, and research on the effects of exercise and sport among refugees is still in its infancy. Thus far, no randomized controlled trials have been carried out in a refugee camp setting, most likely because such trials are complicated by multiple organizational and methodological issues. We highlight some major challenges when carrying out experimental research in a refugee camp. *Method* This report of professional practice is based on systematic observations, individual and focus group interviews, and experiences made in a pre-experimental study, implemented in a refugee camp on the Greek mainland. *Results* The report provides background information about how refugees generally come to Greece, how transit camps are typically managed, which institutions are involved in the camp management, which rules need to be followed by people working in a camp, which countries refugees generally come from, and the conditions in which they live in the camp. We also identify general factors that complicate experimental research in such a setting, and highlight specific issues pertaining to sport and exercise-based intervention trials. *Conclusions* Currently, more people are fleeing their home regions than after the end of the Second World War. This situation calls for a change in the understanding of humanitarian aid. Pure material and technical support must be complemented by public health measures, including exercise and sport programs. Researchers who want to enter this field of research can learn important lessons from our observations.

## 1. Introduction

In addition to the physical toll that becoming a refugee can take, being forced to flee one’s country frequently engenders great psychological distress. Many refugees are faced with difficult living conditions, economic hardship, discrimination, social exclusion, and exploitation. Additionally, prior to their escape, this population has often been exposed to severe mental and physical strain due to war, violence, political and religious persecution, poverty, imprisonment, or torture [1].

It is now well established that refugees have a considerably higher prevalence of psychopathological disorders such as post-traumatic stress disorder (PTSD), depression, or anxiety disorders [2]. As defined in the ICD-10 (International Classification of Diseases, 10th edition), PTSD is the result of a long-lasting or pervasive strain due to a devastating natural disaster, combat, severe accident, witnessing a violent death, torture, terrorism, or rape. The mental disorder is a direct result of the trauma experienced. Trauma is a perceived discrepancy between the life-threatening situation and individual coping mechanisms [3]. According to the ICD-10 definition, PTSD is characterized by the following symptoms: recurring and uncontrollable memories or dreams involving the traumatic event (flashback); feelings of numbness and emotional blunting; social isolation; avoidance of activities and situations reminiscent of the trauma; acute outbreaks of anxiety, panic, or aggression; fright reactions; insomnia; persistent anxiety; and depressive symptoms. In addition, suicidal thoughts and excessive substance use may occur. For some of those affected, the disorder may persist for years and lead to a permanent personality change.

The risk of a chronic course is particularly high for refugees. Keilson [4] used the term “sequential traumatization” to refer to the particular circumstances of a distressing journey from the country of origin, compounded by uncertainty and harsh living conditions, as well as the likelihood of further upheaval upon arrival. Among refugees, traumatic experiences in combination with the long drawn-out asylum process, a sense of being trapped and a fear of deportation often result in feelings of apathy or resignation [5]. This may explain in part why physical inactivity is a common phenomenon among refugees and immigrant populations [6,7,8]. Although prevalence estimates are scarce, Madsen et al. [9] have reported that nearly 50% of all asylum seekers in Denmark were tortured, and at least two in three refugees met the ICD-10 criteria for PTSD.

These disorders are often accompanied by somatoform disorders and pain [10]. Recently, Médecins Sans Frontières [11] have criticized the lack of early interventions as a key problem, particularly as many asylum seekers suffer from untreated mental disorders that are subsequently difficult to cure. Despite this, in the field of psychiatry, little attention has been paid to the treatment of traumatized refugees [12].

Exercise and sport have been successfully employed to treat a wide range of psychiatric disorders [13], and are considered essential to human wellbeing [14]. Although the UNHCR (United Nations High Commissioner for Refugees, Geneva, Switzerland) recognizes the potential of exercise and sport as a peace-building measure in refugee camps [15], and reviews have shown that increased physical activity has positive effects on traumatized individuals [16,17], very limited evidence is available regarding the potential of exercise and sport as part of the treatment of refugees suffering from PTSD symptoms [18]. In a study with 36 refugees with PTSD symptoms living in Germany and Switzerland, Liedl et al. [19] showed that, following the intervention, refugees who participated in 12 weeks of biofeedback-based cognitive behavioral therapy (CBT-BF) in combination with physical activity (including mixed activities such as endurance, strength, and flexibility training) reported an improved capacity to cope with pain compared to CBT-BF alone or a waiting-list control condition. In a study with refugees with PTSD symptoms living in Denmark, Stade et al. [20] reported high acceptability, compliance and satisfaction with basic body awareness therapy (BBAT). Most importantly, traumatized refugees indicated that BBAT helped them to relieve pain and tension, bring peace of mind and body, and to improve their sleep [9]. Similarly, Xin et al. [1] observed positive effects on mental health outcomes among Bosnian refugees living in the USA after they had participated in 12 weeks of regular physical activity. Nevertheless, improvements were also found in the control group (who received general information about strategies to improve mental health), and thus no statistically significant differences between the intervention and control group were found at follow-up.

In a recent one-group pre-test/post-test study with 38 male refugees living in a Greek refugee camp, our research group showed that regular participation in an 8-week sport and exercise program has the potential to positively impact on a variety of mental health outcomes, including post-traumatic stress symptoms, depressive symptoms, anxiety symptoms, health-related quality of life, perceived fitness, handgrip strength, and cardiorespiratory fitness [21]. Refugees who participated more often in the exercise program had more favorable scores at post-intervention, after controlling for baseline and potential confounders. Nevertheless, the findings of our study must be interpreted with caution because our limited resources meant that a randomized controlled trial (RCT) could not be implemented; consequently, there was no random assignment of participants to an intervention or (waiting-list) control condition. Although our study showed that a sport and exercise intervention is feasible in a refugee camp setting, we acknowledge that RCTs are required to advance the empirical evidence base in this field of research. Nevertheless, scholars need to be aware that multiple environmental constraints can complicate the implementation of such studies. This is particularly true for studies taking place in transit camps, in which refugees live before they are granted asylum, before they are relocated to other countries or before they are returned to their home countries.

### Goals and Structure of the Present Report of Professional Practice

On the basis of our experiences in the study presented above, the goal of the present report of professional practice was to describe in more detail the daily life conditions of refugees living in (Greek) transit camps, and to identify possible issues that may complicate the implementation of scientific research in general and sport and exercise intervention trials in particular. We believe that researchers can learn from our experiences, and, as a consequence, be able to anticipate some possible pitfalls that might be critical for the successful implementation of an RCT. Although detailed information about the setting in which our study took place is provided elsewhere [22], the most important cornerstones are summarized in Table 1. This basic information is helpful to put our report into perspective.

The present report is structured as follows: We first give some background information about how refugees generally come to Greece. We then explain how Greek transit camps are typically managed, and which institutions are involved in the management of these camps. We also address the issue of whether specific rules need to be followed by people working in a refugee camp, before we briefly report on which countries refugees generally come from, and conditions in which they live in the camp. We then discuss some general factors that might complicate the implementation of experimental research in such a setting, before we highlight some specific issues pertaining to sport and exercise-based intervention trials. Further aspects that are addressed in the present report of professional practice are acceptability and subjective benefits, specific requirements for coaches, sustainability, and some critical reflections on the status of exercise and sport within refugee care.

## 2. Methods

The present report of professional practice was based on the experiences made by the first author of this article (F.K.) during two extended stays in Greece. From April to October 2016, the first author spent 3 months (in total) in different refugee camps on the Greek mainland, where he worked as a volunteer for Northern Lights Aid (see http://www.northernlightsaid.org). His main tasks consisted of craft activities, sorting and distribution of relief supplies, and the implementation of activities as a sport coach. During this time, he made the necessary arrangements to carry out the intervention study described above. From August to October 2017, he spent another 3 months in the Sinatex refugee camp, where he implemented the exercise and sport intervention. The insights presented in this paper are based on occasional observations made in several refugee camps, personal interviews with different stakeholders from non-governmental organizations (NGOs; camp management, medical aid organization, other volunteers), and the refugees themselves. The interviewees were approached directly, and asked whether they would be willing to take part in an interview. In order to not overburden the refugees with the numerous surveys, the interview was attempted to be short and lasted around 30 min. None of the individuals approached refused to participate. In total, one interview was carried out with camp management staff, two with people from medical aid organizations, four with other volunteers, and seven with refugees. Because the in-depth interviews were conducted in English, only refugees with moderate to good English skills were selected. Interviews were organized in a private space (outside, in the shade, compartment, away from the other camp residents) under calm circumstances during different times of the day. For the different groups of interviewees, different guides were developed to carry out semi-structured interviews (including four to six questions). Examples of questions asked to refugees were: What does your everyday life in the camp look like? Which factors burden you most during everyday life in the camp? Which factors prevent you from participating in exercise and sport activities? Which exercise and sport activities did you pursue before you left your home country? At the beginning of the interview, each interviewee was asked to briefly introduce him-/herself. The interview questions were then used as a starting point; all interviewees were asked to talk freely and in detail, and ask their own questions if necessary. Owing to the uncertain legal and political status that is a part of life as a refugee, the majority of refugees preferred not to be audio-taped during the interviews. We therefore decided to take only handwritten notes during the conversations. The key findings from the interviews (and observations) were recorded in a diary.

Furthermore, four separate focus-group discussions were organized with the camp residents to get insights into the daily life in a camp and to learn more about the residents’ preferences regarding the contents of the sport and exercise program. Male camp residents were approached by F.K. during the first week of his stay in the camp in August 2017, when they were in public areas of the camp and invited to attend a group at a specific time and place. They were informed about the goal of the focus group discussion, and encouraged to tell their friends. The focus-group discussions were carried out outdoors in the evening hours, under calm and familiar circumstances (e.g., having a glass of tea and snacks) with four to five participants in each focus group. The conversations were held in English. This time, however, non-English speaking refugees were also included (with other refugees acting as translators). This procedure allowed us to have discussions with people from different countries of origin and educational background. The interview had a less formal character and seemed to encourage the participants to open more up. A question was asked, and each participant had the possibility to respond to this question. Afterwards, a conversation was initiated by discussing the different perspectives. For each focus group discussion, four to five questions were prepared. Typically, the discussions ended after 50 to 60 min. Again, we did not audio-tape the discussion, but handwritten-notes were taken. After each focus group, the key findings were recorded in the first author’s diary.

Prior to the beginning of our intervention study (August to October 2017), all documents were submitted to the Ethical Review Board of Northwestern Switzerland and Central Switzerland (EKNZ, project-ID 2017-01115, 6 July 2017). Because the study took place in Greece, the EKNZ required approval of a local ethics committee. Therefore, ethical approval was sought and granted (8 August 2018, no project number) by the institutional review board of the medical association responsible for primary care at the camp where the intervention took place (Kitrinos Healthcare, London, UK). All procedures used in the intervention trial followed good clinical practice and were in line the ethical principles defined in the 1964 Declaration of Helsinki and its amendments. Furthermore, the first author of this article signed the “Code of conduct” of the Danish Refugee Council [22], which is composed of several ethical principles that must be respected by people working in the camp (see Section 3.1.3. for more details).

## 3. Results

### 3.1. Background Information about Greek Refugee Camps and Their Residents

#### 3.1.1. How Refugees Come to Greek Refugee Camps

Most refugees reach the Greek (transit) refugee camps via Turkey. From there, they can reach the nearby Greek islands such as Lesbos, Chios, Samos, or Kos after a life-threatening crossing in rubber dinghies. Because of the border closure of Macedonia in March 2016, a large number of refugees are stuck in Greece. With no way to travel or return, they sit in one of the many camps run by the Greek government. Incoming refugees are registered on the islands and after a while are relocated to a camp on the mainland. Since the EU–Turkey Agreement of 2016, which among other things provides for the repatriation of refugees from the islands to Turkey, the refugees on the islands are increasingly being held back. This is causing refugees to increasingly reach Greece via the land bridge, which is not necessarily less dangerous due to the crossing of the (underestimated) border river Evros. Arriving on the mainland, the refugees are taken to one of the conurbations in Athens or Thessaloniki and assigned to a specific refugee camp.

The camps are located in former military bases, empty factories, unused quarries, or airfields. Although some camps are located right in the center, others are far away and can only be reached by long bus trips. Some camps were built right next to the beach and thus offer natural opportunities to the refugees to occupy themselves. Most camps located in industrial or agricultural areas do not offer this option. Moreover, there are substantial differences in terms of equipment among the different camps. Whereas the refugees live in containers with kitchens and air conditioning in some camps, in others, they live in tents or compartments separated by thin walls. Refugees generally receive a sleeping place, basic medical care, and a monthly allowance of 150 euros per person. The money must cover any costs incurred, for example, for food, clothing, mobile phone bill, cigarettes, or the bus ticket to the city.

In the camps, refugees wait for an appointment with the Migration Office to make their asylum application. They then wait again until their application is considered and a decision is taken. Due to the high number of refugees, this process can take over a year, depending on the personal situation. The Migration Office will then decide whether the refugees are granted asylum in Greece, whether they are allowed to reunite their families with other family members in Europe, or whether their asylum application is rejected, leading to repatriation.

From their arrival in Greece, most of the refugees spend several months to years in one of the camps. Their legal status does not allow them to pursue gainful employment. Scarce financial resources also limit their leisure time activities and their freedom of movement in general.

#### 3.1.2. How Refugee Camps Are Managed in Greece

Refugee camps in Greece are usually subordinate to the Office of Migration and run by the military. As a result, several soldiers are permanently present in the camps. In addition to the soldiers, some police officers are also stationed in the camp. They are responsible for security, and carry out checks at the entrance. Outsiders may enter the camp only with a permit. Access is also granted if a person is employed by a non-governmental organization (NGO) working in the camp and registered with the Migration Office.

There are usually several institutions and organizations working closely together in a refugee camp. To provide an example, the camp where we conducted our study was managed by the Danish Refugee Council (DRC). The employees of the DRC are contact persons of the refugees in administrative matters. They also take on various other tasks, such as equipping the living quarters and organizing smaller activities.

If someone wants to initiate a (sport or exercise) program in the camp, this must first be approved by the DRC. In the camp presented in our study, the NGO “Kitrinos” is responsible for medical care. Kitrinos consists of voluntary working doctors, nurses, and midwives. Refugees are treated in a container equipped as a clinic. A physiotherapist from Kitrinos looks after the refugees’ physical ailments twice a week and organizes a weekly group session targeting back and neck pain. Also stationed in the camp is “Solidarity Now”. Solidarity Now conducts about three hours of playful activities with children in the mornings. In the afternoons, they occasionally organize board games for the men. Solidarity Now also employ the only psychologist in the camp. Outside the camp, the NGO “Bê Sînor” has acquired a piece of land and set up a cultural center. The cultural center acts as a meeting place, with opportunities for sitting and strolling, in which different activities are offered depending on the volunteers present. These include English lessons, women’s groups, music classes, gardening, children’s activities, and drawing and reading lessons. A football field is located behind the cultural center. However, this is not used because of the poor condition of the goals and the playing surface. The last NGO on the ground was “Team Bananas”, which distributed bananas twice a week and sporadically organized a football game. Neither Bê Sînor nor Team Bananas had access to the camp, so their activities were primarily outside the camp.

In summary, cooperation with a local NGO is essential for scholars who want to conduct research of any kind in a refugee camp. The cooperation is not only a basic requirement for access to the camp, as NGO employees are aware of the current situation and individual processes within the camp. Having access to this knowledge will help researchers to react to current events early and flexibly. In addition, researchers may benefit from personal and administrative support provided by the NGO.

#### 3.1.3. Rules for People Working in a Greek Refugee Camp

People working in refugee camps usually abide by a code of conduct, such as that of the Danish Research Council [22]. This code defines certain rules of conduct that all employees must adhere to. This includes respecting cultural specificities such as adequate clothing or behavior. All persons in the camp should be treated with dignity and fairness, without favoring certain individuals. A professional and emotional distance should be maintained towards the refugees, although this can be difficult to combine with an empathic approach. Due to the disadvantaged situation in which the refugees are located and the unequal balance of power, no gifts from refugees may be accepted, as this could bring the recipient into a conflict of interest or debt. The code of conduct also requires employees to discreetly handle entrusted information and not to disclose it, unless there are indications of a specific risk to the health and wellbeing of the person.

#### 3.1.4. Countries Where the Refugees Come From

In Greek refugee camps, the inhabitants are typically very mixed (including people from Syria, Iraq, Afghanistan, Iran, Palestine, etc.). Due to relocations and new arrivals, the number of camp residents fluctuates strongly. This is particularly the case in detention centers where refugees are first admitted. This is important to mention because heterogeneity in terms of language and culture are key challenges that researchers are faced with when carrying out research in Greek refugee camps.

#### 3.1.5. Everyday Life in a Refugee Camp

With the exception of a few highlights, everyday life in refugee camps is characterized by a high degree of monotony. In the camp presented here, the remote location offered little incentive for activities. A lack of recreational opportunities serves to increase the inactivity of the refugees. To pass the time, they frequently use their mobile phones in order to follow the situation in their home country, make phone calls with family or friends, watch videos, play online games, or try to learn the English language through apps. Often the refugees just sit together and talk over a cup of coffee or tea. Cigarette consumption is frequent and widespread. Some refugees occasionally walk around the nearby area. Other employment opportunities are household chores and cooking meals.

In the camp where our study took place, once a week a bus arrived to take the residents to a large supermarket and picked them up again. A few residents had established their own small business in the camp by cutting hair, selling goods or making iced coffee for a small fee, sometimes free of charge. In the evening, the men often played cards. Periodically, some of them also organized a football game late in the day, sometimes not until midnight, on a narrow strip of concrete between the toilet and shower containers next to the factory building where the other refugees slept. Occasionally, the residents were able to travel by bus to Thessaloniki. Young men in particular made use of this offer (especially in the first two weeks of the month after receipt of the monthly pocket money). In addition to Thessaloniki, some residents travelled to other camps or to Athens to visit refugees living in other camps or simply to gain some distance from the camp routine.

#### 3.1.6. Burdens Refugees Are Confronted With

In the camp where we operated, the organization responsible for the medical care (Kitrinos) estimated that 80% of clinic consultations concerned the mental wellbeing of refugees. As already described above, the everyday life of the refugees is often characterized by monotony and melancholy. This is reflected in the overall mood of the refugees and in the camp atmosphere. Due to the monotony, the refugees have plenty of time to ruminate on the traumatic events experienced before or during their escape. Some experience flashbacks and scream during sleep. Fears of the future and post-migratory stress factors represent an additional burden. Many refugees fear for relatives and close friends. In the best case, they are still in direct contact with them. In other cases, the contact is broken off and information about recent incidents in the affected area and related clues as to the wellbeing of the family are only obtained through social media. In addition, there is a lack of perspective and uncertainty, which is linked to the wait for the asylum decision and a threat of rejection.

In the camp presented here, due to clashes, attacks, or thefts within the camp, the mood among camp residents was characterized by excessive vigilance, mistrust, and concern. These factors seemed to stay in the way of dealing with traumatic experiences. A large proportion of the residents also suffered from both mental and physical fatigue. The difficult climatic conditions during the summer months (high heat) also affected the sleep and day and night rhythm of the refugees. In this region of Greece, summer temperatures are quite high (about 33 °C during the day), and thus the buildings in which the refugees live often heat up during the daytime, making it very warm even at night. The younger camp residents often stayed awake at night until the early morning hours and slept only a few hours during the day or not at all. Due to the close-fitting compartments, this often meant that other families were kept awake. Even the occasional midnight football game contributed to the impairment of the sleep of the other camp residents. Accordingly, sleep complaints were often reported by the refugees, and many of them felt that they are too tired and too exhausted to be physically active during the day. However, fatigue can also be attributed to other factors such as lack of drive through depressive moodiness or diet-related iron anemia [23].

Moreover, we observed that the frequent coming and going of refugees challenged the development of a sense of community in the camp. Sometimes, refugees were the victims of petty crimes carried out by other camp residents and even their own roommates. For fear of revenge, however, they did not always turn to the police. Under these circumstances, many refugees found it difficult to rest. In the small and open-topped compartments, it was difficult to create a sense of personal space or escape the hustle and bustle. The strong ethnic mix combined with the great mental stress also had the potential to fuel conflicts; on a number of occasions, altercations occurred between different ethnic groups over ostensibly trivial matters. Folding knives were carried by some young men, and misused in conflicts. This left many refugees with a sense of fear and insecurity.

Additionally, we identified confrontation with racism and the feeling of being unwanted as further relevant stress factors. Although the refugees were warmly received in some places, they met a harsh climate elsewhere. This can be through discriminatory acts in everyday situations with people from the region. For example, during our study, it was repeatedly observed that the bus to the city (Thessaloniki) did not stop for the refugees waiting at the bus stop of the camp.

Finally, health problems can also result from a one-sided diet. The refugees are responsible for shopping and preparing food themselves. Lack of experience in cooking and inadequate knowledge about a healthy diet pose challenges for some refugees. Although some refugees are well fed, the diet of others is composed of nutrient-poor, high-fat, and carbohydrate-rich foods. We also observed that a number of refugees preferred to save the money for expected upcoming difficult terms or invest the scarce money in alcoholic beverages and/or cigarettes rather than in more expensive foods such as fruits, vegetables, or dairy products. Certain refugees also consumed drugs, with few camp residents using drug trafficking as a source of income.

### 3.2. Factors Complicating the Implementation of Experimental Research

#### 3.2.1. Permission and Ethical Clearance

In order to carry out empirical research in a refugee camp, long-term planning is needed. As mentioned earlier, the refugee camps fall within the jurisdiction of the Office of Migration and run by the military. However, the actual management of the camp is often the responsibility of another institution that works closely together with different NGOs.

In the best case, the approval of all these institutions is available before the start of an empirical study. However, this process can be somewhat lengthy, as it is sometimes difficult to reach the right person within these institutions. In addition, it is important to inform the organizations in a camp about a planned study in good time and to coordinate the planned intervention with existing activities so that there is no competitive situation. The institutions working in a camp may also be involved in carrying out a study; alternatively, their cooperation may be required in carrying out certain tasks, and this must be clearly communicated. In this context, cooperation in the form of a collaborative research agreement is advisable, in order to clarify the rights and obligations of the involved partners. Because only NGOs registered in Greece have access to the refugee camps, cooperation with a partner institution is essential.

Sufficient time must also be allowed for the ethical review of a study. This is particularly true if the study is conducted by a foreign institution. In this case, the study should first be examined by an ethics committee in the home country of the leading research group. This raises the question of which institution in Greece conducts the ethical review. As this is not always obvious to foreign researchers, it is advisable to work with a Greek partner institution (e.g., university) that is familiar with local customs and practices. If approved by the responsible ethics committee(s), the study should be registered in an international trial registry. If research equipment is imported, it is necessary to familiarize oneself with the national import regulations.

#### 3.2.2. Informing Study Participants

Before embarking on a study, it is important, in consultation with the partner institution in the camp, to look for suitable strategies for making the refugees aware of a planned study. There are various ways to get in contact with the refugees. This can involve the distribution of food parcels by a study employee, or the initiation of some low-threshold sports activities such as volleyball or football, among other activities. Regular presence, as well as a willingness to approach the refugees and seek personal contact, are key to recruiting a sufficient number of participants.

Great importance should be attributed to adequately informing the study participants. Refugees are in many ways a particularly vulnerable population. On the one hand, the camp residents are in a dependency relationship. Their future depends on the asylum decision of the competent authority; consequently, much is at stake for them. On the other hand, the refugees are in a crisis situation; many of them are traumatized and suffer from mental and physical complaints. When carrying out an empirical study, it should therefore be expected that there will initially be mistrust over the use of the collected data. Sufficient time should be provided to inform the refugees about aspects such as voluntariness, confidentiality of data, benefits and risks associated with study participation, consequences of dropping out of the study, and data security. Intensive educational work is therefore also of central importance to counteract falsified data. Refugees who are in the asylum process may, for example, assume that they have a better chance of obtaining a residence permit if they have high mental symptoms.

Beyond oral information provided by a research officer, all information should be given in writing to the study participants. Because the refugees of a camp usually speak different languages, sufficient financial resources should be allocated for interpreting and translation work. In the best case, the documents are available in every language, and at least one interpreter is available who is familiar with all spoken languages.

In case of limited resources, refugees with good English skills may also assist the investigator in informing potential participants about the study. It should be noted, however, that working as a translator can be very tiring. Because most eligible refugees themselves are physically and mentally stressed, care must be taken to ensure that the interpreters are not overloaded with work. Their current state of mind can also affect the motivation and quality of the translation. Our experience shows that most refugees with advanced English skills like to work as interpreters. They perceive this work as a meaningful task. This also has a positive effect on their status among the refugees in the camp. However, this results in a potential conflict with the code of conduct, as all refugees are to be treated equally in the camp. Collaboration between researchers and interpreters, however, inevitably leads to more time being spent with certain people.

#### 3.2.3. Selection of Instruments

Ideally, any instruments used in a study will be validated internationally, and for different languages and cultures. If an instrument is not available in a specific language, it should first be translated in a forward-backward process [24]. In other words, an instrument should be translated into the appropriate language by a native speaker, then back-translated into English by an independent person. In case of deviation, the translation must be improved and the process described above must be repeated, if necessary. Then, the instrument should be tested within a small number of refugees. This process can take several weeks. The selection of relatively short instruments is also recommended, as many refugees might find it difficult due to mental stress to concentrate for a longer period of time.

#### 3.2.4. Data Assessment

In a study with refugees, it should be expected that the collection of data will take more time than is usual with other study populations. In our study project, refugees had little prior experience with empirical data assessments. Moreover, many refugees may not have been involved in questionnaire-based research, and need help completing the questionnaires. Also, one should assume that illiteracy can occur among refugees, and ensure that an interpreter or study employee is prepared to administer the questionnaires orally. Furthermore, simultaneous assessment of a larger number of participants can be difficult in a refugee camp due to the lack of necessary infrastructure (such as suitable premises with enough tables and chairs). Overall, it is advisable to carry out the data collection in a one-to-one format or in small groups. In case of performance tests, such as of cardiorespiratory fitness or cognitive function, it is recommended to demonstrate the test in advance to prevent misunderstandings.

#### 3.2.5. Examination of Longer-Term Effects

In order to ensure that an intervention triggers the expected effects on the participants, and to examine the sustainability of the intervention, RCTs should include a sufficiently long intervention period, and at least one follow-up assessment. However, as will be discussed in the next section, multiple factors contribute to the fact that a relatively high dropout rate is to be expected in studies with refugee camp residents. Researchers should therefore be prepared to find ways to deal with intention-to-treat effects. Furthermore, refugee camps themselves are not stable institutions. For instance, the camp in which our study took place (Sinatex) was closed in February 2018 (only 3 months after our data assessments were complete), and refugees were relocated to the Lagkadikia camp. Thus, higher-order political decisions can have a major impact on experimental studies, and jeopardize the successful implementation of well-planned intervention trials.

#### 3.2.6. Obtaining Information via In-Depth Interviews and Focus Group Discussions

Obtaining information via in-depth interviews and focus group discussions (e.g., regarding the preferences of the refugees with regards to intervention measures or the perceived benefits of an intervention) can be very valuable in order to increase participants’ ownership of the contents and structure of the program, as well as fostering self-determination. In our case, it was a useful tool with regards to choosing activities and intensity levels that would be attractive to participants, as well as reflecting their existing knowledge and skills. However, overcoming language barriers is a significant challenge when collecting these kinds of data. Focusing on participants with advanced English skills might introduce a bias (e.g., focus on individuals with higher educational levels). Although working together with a translator might be a viable option to solve this issue, this procedure makes the interviews even more time-consuming, leading to a less direct conversation. Moreover, as our experience has shown, many refugees are reluctant regarding the use of audio-taped material. As a consequence, transcription, organization, analysis, and reporting of the results of in-depth interviews and focus group discussions might be complicated.

### 3.3. Factors Requiring Attention When Implementing Exercise and Sport Intervention Trials

#### 3.3.1. Recruitment of Study Participants

For several reasons, recruiting refugees for an exercise and sport program was one of the biggest practical challenges. It should first be noted that recruiting refugees requires care and sensitivity. As noted above, many refugees suffer from severe psychiatric disorders.

When recruiting participants for an exercise and sport intervention, the four-phase model by Ehrenreich [25] can provide guidance. This model highlights that different needs dominate in different phases. The first phase, the so-called rescue phase in the first week after the disaster is characterized by a struggle for survival. In the second phase, which ranges from the second week to four months, the focus turns to the satisfaction of basic needs. This is followed by the disillusionment phase, which can last from the fourth month to two years. In this phase, grief and depression are likely to be experienced. The final phase, which can last two years or more, involves overcoming existing impairments. On the basis of this model, it appears that early participation in an exercise and sport program can drain the resources that are needed to work through difficult emotional experiences. Those affected need to be given time to deal with the situation, as well as the opportunity to mobilize their own resources. However, delaying participation may result in a person’s physical condition deteriorating to such an extent that it becomes increasingly difficult for them to return to a physically active lifestyle.

In our study, some refugees showed great interest in the exercise and sports program and appeared on time for training, whereas others were interested, but unable to engage in exercise due to war-related injuries such as gunshot wounds, or untreated foreign bodies such as bomb shrapnel or shattered bones. Some refugees made it clear that they were not interested in such a program. However, it must also be noted that many participants found it difficult to reach a clear decision. Several refugees said they wanted to be more physically active, but would miss training sessions despite promises to attend.

Providing an incentive can further help with the recruitment of participants with little prior experience of exercise and sport. However, the provision of monetary incentives may violate the code of conduct in some camps, and could be seen critically from an ethical point of view. In our study, we decided to offer a bar of chocolate to all participants after each data assessment (pre-test and post-test). Our experience showed that such a small incentive was indeed helpful in increasing refugees’ motivation to engage in the study.

#### 3.3.2. Selection Bias

In a study with refugees, many factors can contribute to a selection bias, including age, gender, education, socioeconomic status, and prior participation in exercise and sport. In our study, individuals who had been away from their country of origin for longer showed greater willingness to participate in the exercise and sport program than those who had undertaken their journey more recently. This may be because the latter primarily pursue the goal of reaching Central Europe and, accordingly, are more involved in the onward journey. Refugees who had been on the road for a while may have come to terms more with the idea of being stuck in Greece, and thus showed greater interest in getting involved in a new life situation and pursuing active leisure time. As participation in research was voluntary, it was impossible to control for all of these factors. However, in order to judge whether the selected participants were representative of the overall refugee population, it seemed important to assess their basic social and demographic information.

#### 3.3.3. Medical Precautions and Adverse Events

An exercise and sport program involving individuals with physical and mental disorders requires special precautions. First, it is advisable to employ the Physical Activity Readiness Questionnaire [26] to determine possible contraindications for participation in moderate-to-vigorous physical activity. In case of such indications, cooperation with a medical-oriented NGO working in the camp can be helpful in conducting physical aptitude assessments, treating injuries that occur during training, or resorting to medical expertise if necessary. Such precaution measures are particularly important if the testing includes a maximal fitness test and/or high-intensity exercise and sport activities. Researchers should also establish emergency plans in case of (unexpected) adverse events. This is especially important when a camp is located in a remote area. As adverse events are more likely to occur in traumatized refugees than in other study populations, specific attention needs to be paid to the development of plans for collecting, assessing, reporting, and managing adverse events and other unintended effects of the intervention.

#### 3.3.4. Selection of Suitable Contents

The selection of exercise and sport activities presents a further challenge in refugee populations. Our experience shows that the heterogeneous composition of camp inhabitants leads to extremely heterogeneous preferences and demands from a training program. This concerns not only the type of activities, but also the intensity of training and the role and involvement of the coach. For instance, in our study, some participants liked being cheered on by the coach, whereas others felt disturbed because they were reminded of their military service.

In addition, some sports such as football enjoy general popularity. However, football, if wrongly implemented, can fuel conflicts and provoke violence, exclusion, and nationalism [15]. If people from different cultures participate in a program, ethically mixed teams can defuse the situation. Nevertheless, in general terms, our recommendation is that exercise and sport programs should be geared to the sociocultural background and the individual needs of the refugees. Moreover, to maintain the motivation of the participants, it seems advisable to implement a varied program design, and to give participants some choice in the activities. In our study, we chose to extend the training from three (Monday, Wednesday, Friday) to five (Monday to Friday) days and to complement it with a broader range of activities. Although on Monday, Wednesday, and Friday, the focus was on strength and endurance training, on Tuesday and Thursday we offered either football, volleyball, boxing, or partner acrobatics. This gave participants a certain choice to engage in activities they liked.

Finally, an exercise and sport program with a high proportion of traumatized refugees should be considered as more than mere “fitness training”. Thus, a training program tailored to this target population should have a particular focus on strengthening participants’ psychosocial resources. This includes enabling mastery experiences, promoting body awareness, distraction from current issues through challenging tasks, building shared values (such as respect, fairness, non-violent conflict resolution), and empowering the community at large.

#### 3.3.5. Investigator Effect

In order to provide the above (positive) experiences as a part of an exercise and sport program, a trusting relationship between the participants and the coach is necessary. However, this always entails the risk of an investigator effect. In order to avoid such an effect in scientific studies, one possibility would be to offer an equal amount of social activities for non-participants (e.g., board games, table football, darts) in order to control for social contact. Moreover, to avoid issues with social desirability, it should be ensured that the data assessment is carried out by blinded personnel who were not involved in the implementation of the training program.

#### 3.3.6. Scheduling

When implementing an exercise and sport program, it is important to ensure that the training times are integrated into refugees’ existing daily routines. Muslim refugees, for example, have to take into account prayer times, and religious customs and festivals must also be considered, such as the fasting month of Ramadan, the festival of fasting (Eid al-Filtr), or the sacrificial festival (Eid al-Adha). These customs/celebrations may restrict participation in an intervention trial. In the current study, a particular Muslim festival meant that training was suspended for four days; after this, a number of participants had difficulties returning to regular exercise.

In our own study, 19:00 proved to be a suitable time for the start of the training. At this time, most of the participants had already eaten and there was enough time before evening prayer. In addition, the temperatures were no longer prohibitive.

#### 3.3.7. Communication between the Coach and Participants

As mentioned previously, language barriers are a central problem in the implementation of an exercise and sport program in a refugee camp. This not only complicates data assessment, but also the implementation of an intervention. Although much can be compensated by non-verbal communication (showing exercises, using facial expressions and gestures), the lack of language skills often hinders deepened conversation. This makes it difficult, for example, to establish a personal relationship with the training participants, to communicate shared values, to take team-building measures or to obtain feedback. Limited language skills also complicate the motivational work of the instructor. In instances where language barriers pose a significant obstacle, simple activities (for example, football, boxing, strength and endurance training) that do not require detailed explanation are recommended.

#### 3.3.8. Sports Equipment and Infrastructure

Refugees often do not have adequate sports equipment. Many refugees do not have sports shoes, so they train either in inadequate shoes or barefoot. The training program can also be hampered by the lack of suitable infrastructure (e.g., no flat training ground, no covered area). As part of our project, only a small asphalt site was available next to the factory/residential building. The training-related noise made some other campers feel disturbed, although a number of participants joined the program after seeing it in close proximity.

Typically, resources for the procurement of exercise and sport material are scarce in refugee camps. However, usable sports equipment can be developed by simple means. For example, in our project, we used plastic bottles filled with sand as dumbbells or big water cans as kettlebells.

#### 3.3.9. Dropout and Compliance

Issues related to (limited) compliance represent another significant problem area. In the context of scientific studies, a certain dropout rate is to be expected when determining the sample size. In the case of studies with refugee populations, reasons for dropout are government-induced relocation, attempts to travel independently to Central Europe, a deterioration in health, or loss of interest.

Nevertheless, several other factors can have a negative impact on participants’ compliance. In our study, visiting friends in other camps or appointments with authorities in Athens resulted in several participants having to miss out on training sessions. In addition, at the beginning of the month (after receiving their allowance), the young men often travelled to Thessaloniki (the nearest major city). This made it difficult for them to attend the training regularly. Although fathers were among the more regular participants in the intervention program, they often found it difficult to reconcile exercise with their family responsibilities (such as taking care of children).

In addition, drugs were used by some participants of our study. In some cases, this meant that individuals had to be excluded from certain sessions because they appeared to be training under the influence of drugs. General worries such as heartbreak were also reported as obstacles. Furthermore, our experience showed that the everyday life of the camp could have a strong impact on the mood of the refugees. Due to the dense population conditions, inhabitants witnessed every incident that occurred. As a consequence, events such as suicide attempts or abortions within the camp had a substantial effect on the residents’ mood and motivation. In another instance, a conflict between Kurds and Iraqis resulted in a critical event. One participant caught in the middle of the conflict experienced severe mental and physical trauma, which led to him avoiding social contact and, consequently, exercise participation.

In discussions with the refugees, non-attendance at training sessions was mostly attributed to impairments in mental well-being. Many refugees reported that they felt so mentally unsettled that they did not want to start training. Some refugees reported that past experiences would play out again and again in their heads during training. Other impediments described were physical complaints, with back pain being particularly common. Accordingly, the willingness to participate in training depends heavily on fluctuating individual and environmental factors. The weather (temperature) could also pose a significant obstacle to exercising. In addition, bad news from home, family members or the authorities (e.g., negative asylum decision) played a crucial role.

A further issue was the establishment of structure for a group of people who have necessarily existed outside of a structured environment for a long period of time. Although in our study the participants were informed a number of times that training starts punctually at 19:00, often only a fraction were present at this time. A mere reminder of the training proved to be an insufficiently effective measure. Therefore, we initially collected participants by going from door to door. Some said they had forgotten the appointment, others suddenly felt too exhausted, others were asleep, or were preparing dinner. Later, in order to ensure a punctual start, we decided to remind the participants about half an hour before the training and pick them up at their accommodation. Additionally, we decided that at the end of the day, the coach should make a tour of the camp to maintain relationships with the refugees. Exercise and sport were rarely discussed, as a focus was placed on showing interest in each individual and their situation. Often the coach was invited as a token of appreciation and cultural courtesy for tea or dinner. Finally, our experience showed that group cohesion is another critical factor that may have an impact on participants’ compliance. In order to increase group cohesion, we decided to systematically build in partner exercises in the training program and to establish some group rituals (e.g., handshake), which were carried out after each training session.

In summary, achieving a high compliance rate will be a major challenge even in well-planned intervention trials. As multiple factors impact program attendance, it can be expected that compliance rates will vary widely. Therefore, it is important that attendance is monitored thoroughly in order to include this factor as a covariate or moderator variable. If the resources allow it, including a systematic monitoring of intensity-levels and energy expenditure during the training sessions will allow researchers to judge whether or not targeted (moderate-to-vigorous) physical activity levels were achieved.

#### 3.3.10. Critical Events during the Intervention Period

As mentioned previously, there is a high likelihood that refugees will be exposed to further traumatic or critical life events during their stay at a refugee camp. Whereas occurrence of such events cannot be prevented or controlled by the researchers, it is advisable to collect systematic information about such events. This may make it possible to statistically control for these influences as potential confounders when examining the effects of an intervention.

## 4. Acceptability and Perceived Benefits

In our study, the exercise and sport program consisted of 32 training sessions across an 8-week period. As reported previously [21], the mean participation rate across our sample was 7.5 training sessions (*SD* = 6.5). However, participation varied greatly, with a range of 0 to 23 attended sessions. After completion of the study, participants were invited to answer five questions to get insights into the acceptability of the sport and exercise program, using a five-point Likert scale with the following answering options: 0 (no), 1 (rather no), 2 (neither yes nor no), 3 (rather yes), and 4 (yes). In total, 94% of the participants answered the question “I liked to participate in the program” with yes or rather yes. Moreover, 100% of the participants indicated that they felt (or rather felt) better after the training, 89% reported that the training program had (or rather had) a positive effect on their well-being, and 61% felt (or rather felt) that the program helped them to cope with their daily challenges. Finally, 94% answered the question “I would also like to have sport and exercise program in the future” with yes or rather yes.

With regard to perceived benefits, the refugees who commented on the subject assessed the benefits of the sports program differently. Some refugees said the program had helped them become more active physically. Some said that the program brought some variety to their monotonous everyday life. Some also said that the exercise and sport program offered them a perspective; something to look forward to again. One refugee experienced a marked improvement in headache as a result of training, whereas others reported a positive effect on their degree of exhaustion. One refugee stated that he repeatedly felt good about being spurred on by others during training; another refugee believed that he could use the training as a valve to reduce negative energy. Several participants also stated that as a result of their training, they developed better body awareness.

Nevertheless, some participants commented critically on the training program: Some found the activities too strenuous and thus felt overwhelmed during the training. One participant was reminded of his military time in the course of strength training, whereas another criticized the occasionally aggressive play of training partners in football games and wished for a stronger intervention on the part of coach.

## 5. Required Attributes of a Coach

If researchers initiate an exercise and sport program in a refugee camp with ailing and traumatized people, it is important to select a coach who is attuned to the work he/she will be involved in. Enthusiasm for sport is not sufficient. The coach should have (or be willing to acquire) trauma-specific background knowledge. This is necessary to better understand the circumstances of the refugees and avoid potential trigger factors. Stimuli that are linked to trauma can lead to flashbacks, which vary from person to person. Seemingly innocuous moments, such as a walk in a forest, may evoke memories of their traumatic journey, whereas raised voices can be associated with military time and captivity. We also learnt that it is helpful to inform the refugees in advance about the forthcoming activities, in order to create confidentiality, feelings of trust, provide opportunities for participation, and react appropriately to any negative comments or signs given by participants.

The cultivation of functioning interpersonal relationships requires a certain amount of cultural awareness and knowledge about the history, beliefs, customs, and language of the refugees. The coach should have the ability to empathize, and understand the perspectives, behaviors, thoughts, and emotions of refugees. The coach should also recognize that certain events affect the mood of the camp resident, so that he/she can adjust the training program as needed. A coach should be open to input from participants, reflect on his/her own behavior critically, and learn from experience. It also requires a certain self-confidence to work with a culturally heterogeneous group, to actively approach the participants and set clear rules. As mentioned previously, a degree of personal closeness between the coach and participants, and loyalty to the participants are essential in establishing a trusting relationship. However, balance must be struck between relationship building and respecting the code of conduct. For example, if the coach is invited by the participants for dinner, the meal is essentially a gift. Repeated rejection, however, would be rude and affect the personal relationship with the participants. Being confronted with refugees’ personal lives, showing empathy while simultaneously maintaining a measure of professional distance is likely to be perceived as stressful for most coaches. Therefore, it is advisable to select a person who feels able to deal with stressful and emotionally challenging situations. Last but not least, well-developed communicative skills are required to build a community of common values.

## 6. Sustainability

When researchers initiate an exercise and sport program in a refugee camp to assess the effects on participants’ health, the question arises as to how its implementation can be made sustainable. Ending a training program after a study is over is not ideal from an ethical point of view. The goal should be to establish structures that permit the program to continue after all data assessments have been completed. Although there are no hard and fast rules as to how this can be achieved, it is likely that longer-term programs will be led by volunteers. However, very few volunteers have an exercise and sport science background and should, ideally, be trained accordingly. This also applies if peer workers (e.g., refugees with a special interest in exercise and sport) are one day recruited to be involved in the implementation of exercise and sport programs. In an intervention study, however, a collaboration with peer workers could also help to decrease the aforementioned tension for external coaches of empathizing with and keeping a professional distance towards the study participants [27].

According to UNHCR [28], 86% of all refugees are in so-called low-to-middle income countries (LMIC). In terms of sustainability, many organizations therefore use a “train-the-trainer” approach in their initiatives [29]. Training local coaches creates new jobs and ensures the consistency of programs. Development projects in the field of sports can also include the support of local companies for the construction of the required exercise and sports facilities or the production of training equipment.

## 7. Some Critical Thoughts on the Status of Exercise and Sport within Refugee Care

In a refugee camp, even the supply of basic necessities such as food, primary healthcare, or adequate accommodation can pose major challenges. As a result, aid agencies invest their limited resources primarily in meeting basic needs or in projects promoting primary education and self-sufficiency. In line with this notion, Korsik et al. [15] reported that, due to limited human resources and challenging working conditions, employees were unable to actively implement an exercise and sport program in a UNHCR-run refugee camp in Uganda. Generally, the UNHCR implements sports programs through its partners, such as Right to Play. Achieving a comprehensive supply of exercise and sport facilities is therefore likely to remain of secondary importance. This is unfortunate, as exercise and sport programs have the potential to reduce psychopathological symptoms and contribute to an increase in mental well-being. This is particularly important in view of the fact that satisfying primary needs and restoring safety does not always go hand in hand with an improvement in general and stress-related psychopathologies [30,31].

Finally, it is important to notice that more overarching concerns such as sense of justice/injustice (e.g., regarding the politics of the judicial process and disposal of asylum) may relate to trauma and ongoing traumatization of refugees, and that such perceived injustices might offset some of the potential benefits of sport and exercise programs on participants’ mental health. Accordingly, such complicating factors deserve more extensive study in future research.

## 8. Conclusions

In recent years, conflicts, terrorism, and environmental disasters have had a profound impact on global development. Moreover, it is anticipated that in the future, post-conflict situations will last longer. This changing situation calls for a change in the understanding of humanitarian aid. As highlighted by Henley [32], pure material and technical support must be complemented by public health programs. The treatment of traumatized refugees continues to be a large experimental field and an area with many unexplored scientific angles. Systematic research into different treatment strategies is still lacking [12]. The scientific evaluation of exercise and sport programs is also necessary, as aid organizations are required to demonstrate the effectiveness of certain measures. However, research on the effects of exercise and sport among refugee population is still in its infancy. We hope that with the present report of professional practice we are able to highlight some of the major challenges and potential pitfalls when carrying out experimental research in a refugee camp. Our report is based on a personal account and the experiences made in one single study. We therefore acknowledge that not all aspects might be generalizable to other camps or other countries. Furthermore, the administrative handling of refugees in Greece is subject to rapid change. We also emphasize that our study was limited to male participants, and new/other challenges may appear in programs specifically designed for women. Despite these limitations, we feel that important lessons can be learned from our observations. We hope that more researchers enter this field of research in the near future, and that our report will contribute to the implementation of well-designed and well-conducted intervention trials.

## Figures and Tables

**Table 1:** Basic cornerstones regarding the study, on which this report is based.

Study Design	One-Group Pre-Test/Post-Test Design
Duration	8-week intervention from August to October 2017.
Location	The refugee camp was located in the north of Greece (Thessaloniki region), far away from neighboring villages, in a former industrial area. The camp was located at the foot of a range of hills and was surrounded by agricultural landscape. On the other side of the road, there was a lake, which was difficult to access and heavily contaminated by sewage factories. The nearest village with shops was a 40 min walk from the camp and could only be reached via a thoroughfare. From the main road, regional buses ran to Thessaloniki. The travel time was around 60 min.
Camp	The camp was located on an old factory site. In the interior of the factory, slabs of thin compressed wood were used to build individual compartments, inhabited either by one family or up to four single men. The compartments were open at the top, as long as the refugees did not put together a cover made with blankets or tarpaulins. The compartments were equipped with a small fridge, a fan, and beds with a foam mat mattress. Showers and toilets were located in containers outside the factory premises. In a shared kitchen, several compartments shared a cooking area with a plate stove.
Sport and exercise program	Sport and exercise activities were offered three to five times per week for approximately 60 min per session. Activities were chosen according to the preferences of the participants. The program was prepared in advance and presented to the participants to suggest adaptations. However, due to language barriers, the activities had to be simple and easy to understand. The program consisted primarily of the following activities: football, boxing, and a combination of weight and endurance training. Calisthenics, and weight training using old car/truck tires, sand-filled bottles, and big water bottles were core components of the program. However, other activities such as partner acrobatics, volleyball, and short hiking tours were included as well in order to make the program more varied.
Participants	On average, about 200 people reside in the Sinatex refugee camp (including children and adults, and men and women). Of these, 45 male participants volunteered to participate in the study. Seven participants dropped off, and thus did not complete the post-intervention data assessment. Dropouts reported higher levels of pain and sleep complaints. Dropouts also had higher Body Mass Index (BMI) levels. In total, 38 participants (age: Mean (*M*) = 25.5 years, Standard deviation (*SD*) = 7.1; BMI: *M* = 21.8 kg/m^2^, *SD* = 3.2; time since fleeing home country: *M* = 32.9 months, *SD* = 39.4; weeks in camp: *M* = 20.0 months, *SD* = 18.2). In total, 71% of the participants came from Syria, 16% from Iraq, 4% from Palestine, and 10% defined themselves as Kurds. In total, 74% of the participants were Muslims, 3% were atheists, and 23% did not provide information about their religious background.
Participation rate	On average, participants engaged in *M* = 7.5 training sessions (*SD* = 6.5). A total of 18 participants (47.4%) participated at least once per week in the training (or more often), whereas 20 participants (52.6%) had participation rates below once per week.
Instruments	Symptoms of post-traumatic stress disorder: 22-item Impact of Event Scale—Revised (IES-R) Depressive symptoms: 9-item Patient Health Questionnaire (PHQ-9) Anxiety symptoms: 7-item Hospital Anxiety and Depression Scale (HADS-A) Sleep complaints: 7-item Insomnia Severity Index (ISI) Pain: Visual Analogue Scale (VAS) Quality of life: 5-item WHO-5 Index Self-perceived fitness: 1-item measure Cardiorespiratory fitness: 20 m shuttle run test Upper-body muscle strength: Grip strength test Confounders: age, BMI, nationality, religious background, educational background, time fleeing, time in camp

## References

[1] Xin H., Karamehic-Muratovic A., Aydt Klein N. (2017). Examining the effectiveness of physical activity on mental health among Bosnian refugees: A pilot study. Univers. J. Public Health.

[2] Gerritsen A. (2006). Physical and mental health of Afghan, Iranian and Somali asylum seekers and refugees living in the Netherlands. Soc. Psychiatry Epidemiol..

[3] WHO (2005). International Statistical Classification of Diseases and Related Health Problems.

[4] Keilson H. (1992). Sequential traumatization in children.

[5] Sandalio R.N. (2018). Life after trauma: The Mental Health Needs of Asylum Seekers in Europe.

[6] Crespo C.J. (2000). Encouraging physical activity in minorities: Eliminating disparities by 2010. Physician Sportsmed..

[7] Gadd M., Sundquist J., Johansson S.E., Wändell P. (2005). Do immigrants have an increased prevalence of unhealthy behaviours and risk factors for coronary heart disease?. Eur. J. Cardiovasc. Prev. Rehabil..

[8] Gerber M., Barker D., Pühse U. (2012). Acculturation and physical activity among ethnic minorities: A systematic review. J. Public Health.

[9] Madsen T.S., Carlsson J., Nordbrandt M.S., Jensen J.A. (2016). Refugee experiences of individual basic body awareness therapy and the level of transference into daily life. An interview study. J. Bodyw. Mov. Ther..

[10] Sharp T.J., Harvey A.G. (2001). Chronic pain and posttraumatic stress disorder: Mutual maintenance?. Clin. Psychol. Rev..

[11] Médecins Sans Frontières EU Border Policies Fuel Mental Health Crisis for Asylum Seekers. https://www.msf.org/greece-eu-border-policies-fuel-mental-health-crisis-asylum-seekers.

[12] Hetrick S.E., Purcell R., Garner B., Parslow R. (2010). Combined pharmacotherapy and psychologcial therapies for post-traumatic stress disorder (PTSD). Cochrane Database Syst. Rev..

[13] Lam L.C.W., Riba M. (2016). Physical exercise interventions for mental health.

[14] Lederman O., Suetani S., Stanton R., Chapman J., Korman N., Rosenbaum S., Siskind D. (2017). Embedding exercise interventions as routine mental health care: Implementation strategies in residential, inpatient and community settings. Aust. Psychiatry.

[15] Korsik A., Ivarsson V., Nakitanda O.A., Perez Rosas L.R. (2013). Implementing Sports in Refugee Camps.

[16] Rosenbaum S., Stubbs B., Schuch F., Vancampfort D., Fuchs R., Gerber M. (2017). Exercise and Posttraumatic Stress Disorder (PTSD). Handbuch Stressregulation und Sport.

[17] Rosenbaum S., Vancampfort D., Steel Z., Newby J., Ward P.B., Stubbs B. (2015). Physical activity in the treatment of post-traumatic stress disorder: A systematic review and meta-analysis. Psychiatry Res..

[18] Nordbrandt M.S., Carlsson J., Glahder Lindberg L., Sandahl H., Mortensen E.L. (2015). Treatment of traumatised refugees with basic body awareness therapy versus mixed physical activity as add-on treatment: Study protocol of a randomised controlled trial. Trials.

[19] Liedl A., Müller J., Morina N., Karl A., Denke C., Knaevelsrud C. (2011). Physical activity within a CBT intervention improves coping with pain in traumatized refugees: Results of randomized controlled design. Pain Med..

[20] Stade K., Skammeritz S., Hjortkjaer C., Carlsson J. (2015). “After all the traumas my body has been through, it feels good that it is still working.”-Basic Body Awareness Therapy for traumatised refugees. Torture.

[21] Knappe F., Colledge F., Gerber M. (2019). Impact of an 8-week exercise and sport intervention on post-traumatic stress disorder symptoms, mental health, and physical fitness among male refugees living in a Greek refugee camp. Int. J. Environ. Res. Public Health.

[22] Danish Refugee Council Code of Conduct. https://drc.ngo/media/1214238/drc-code-of-conduct.pdf2007.

[23] Bischoff A., Heuss L.T., Kurth E., Hoffmann S., Schneider M. (2005). “A-Care” Gesundheitsversorgung und Kosten von Asylsuchenden in Basel.

[24] Brislin R.W., Lonner W.J., Berry J.W. (1986). The wording and translation of research instrument. Field Methods in Cross-Cultural Research.

[25] Ehrenreich J.H. (2001). Coping with Disasters. A Guidebook to Psychosocial Intervention.

[26] Thomas S., Reading J., Shephard R.J. (1992). Revision of the Physical Activity Readiness Questionnaire (PAR-Q). Can. J. Sport Sci..

[27] McKeown M., Roy A., Spandler H. (2015). ‘You’ll Never Walk Alone’: Supportive social relations in a football and mental health project. Int. J. Ment. Health Nurs..

[28] UNHCR Global Trends. http://www.unhcr.org/576408cd7.pdf.

[29] Gschwend A., Selvaraju U. (2008). Psychosocial Sport Programmes to Overcome Trauma in Post-Disaster Interventions.

[30] Carta M.G., Moro D., Wallet Oumar F., Moro M.F., Pintus M., Pintus E., Bhugra D.K. (2018). A follow-up on psychiatric symptoms and post-traumatic stress disorders in Tuareg refugees in Burkina Faso. Front. Psychiatry.

[31] Vervliet M., Lammertyn J., Broekaert E., Derluyn I. (2014). Longitudinal follow-up of the mental health of unaccompanied refugee minors. Eur. Child Adolesc. Psychiatry.

[32] Henley R. (2005). Helping Children Overcome Disaster Trauma through Post-Emergency Psychosocial Sports Programs.

